# Patient and family perceptions of a discharge bedside board

**DOI:** 10.1016/j.pecinn.2023.100214

**Published:** 2023-09-11

**Authors:** D.E. McMillan, D.B. Brown, K.L. Rieger, G. Duncan, J. Plouffe, C.C. Amadi, S. Jafri

**Affiliations:** a College of Nursing, Rady Faculty of Health Sciences, University of Manitoba, Winnipeg R3T 2N2, Canada; b Health Sciences Centre, Winnipeg R3A 1R9, Canada

**Keywords:** Patient engagement, Discharge board, Acute care, Engagement continuum, Qualitative study

## Abstract

**Objective:**

To explore patient and family perspectives of a discharge bedside board for supporting engagement in patient care and discharge planning to inform tool revision.

**Methods:**

This qualitative descriptive study included 45 semi-structured interviews with a purposeful sample of English-speaking patients (*n* = 44; mean age 58.5 years) and their family members (*n* = 5) across seven adult inpatient units at a tertiary acute care hospital in mid-western Canada. Thematic (interviews), content (board, organization procedure document), and framework-guided integrated (all data) analyses were performed.

**Results:**

Four themes were generated from interview data: understanding the board, included essential information to guide care, balancing information on the board, and maintaining a sense of connection. Despite application inconsistencies, documented standard procedures aligned with recommended board (re)orientation, timely patient-friendly content, attention to privacy, and patient-provider engagement strategies.

**Conclusion:**

Findings indicate the tool supported consultation and some involvement level engagement in patient care and discharge. Board information was usually valued, however, perceived procedural gaps in tool education, privacy, and the quality of tool-related communication offer opportunities to strengthen patients' and families' tool experience.

**Innovation:**

Novel application of a continuum engagement framework in the exploration of multiple data sources generated significant insights to guide tool revision.

## Highlights

• Most reported board-related patient engagement at consulting level of the continuum.
• Orientation and re-orientation to communication tool increased patient engagement.
• Perceived process gaps in tool application led to board-related privacy threat.
• Provider engagement with board supported perceived patient valuing and quality care.
• Patient engagement requires alignment in patient, provider, and organization goals.

## 1 Introduction

Hospital admissions constitute a major factor in the global health economy, particularly for middle and high-income countries [1] with pre-COVID hospital related expenses at 26.6% of total health care dollars in Canada [2]. Unnecessarily long length of stay (LOS) in hospital by patients no longer requiring acute care services is a significant challenge [[3], [4], [5], [6]] imposing economic burden to the health care system [7,8], increasing risk of patients' health and functional decline [[8], [9], [10]], and creating delays in acute care access for others in need. Collectively, these factors make the identification and evaluation of strategies supporting the appropriate movement of patients through and out of acute care a compelling economic and healthcare priority.

Patient flow [4,[11], [12], [13], [14]] including a reduced risk of readmission [12,15] can be supported by effective discharge planning. Essential to this planning, and supporting timely, efficient, and safe patient care and discharge is patient (and family) engagement (PE) [6,11]. Within the patient care context, a review of the literature indicates a diversity of definitions for PE, ranging from a developmental process reflecting a patient's emotional resiliency in navigating their health care role identity [16], to the patient's desire and capability to participate in care in partnership with health care providers to meet their unique care needs [17].

Carman and colleagues [18] offer a Multidimensional Framework for Patient and Family Engagement in Health and Health Care (MFE), that includes the characteristics of a partnered perspective but situates it along a continuum of engagement that ranges from *consultation*, to *involvemen*t, to *partnership and shared leadership*. This continuum of engagement can occur at three levels: *direct care*, *organizational design and governance*, and *policy making*, offering a helpful guide for exploration of PE within the acute care context. Degree of engagement is based on the flow of information between the patient (includes patient and family) and care providers, and the patient's degree of decision-making power and involvement in organizational and policy change. At the direct care level for example, PE might range from patients as recipients of discharge information, to invitations to identify care preferences, to shared treatment and care decisions. Furthermore, the model considers patient, organization, and societal factors influencing engagement.

While research in PE in health research or innovation is robust [[19], [20], [21], [22]], fewer studies, such as these, have explored PE in inpatient care [19,[23], [24], [25], [26], [27], [28], [29], [30], [31]]. Some research, for example, has studied PE with a patient care and discharge planning focus [6,[32], [33], [34]], but within this focus, few studies were found that specifically targeted acute medical and surgical adult inpatients [[34], [35], [36], [37], [38]]. Bedside visual tools, and specifically whiteboards, are a prevalent category of communication strategies aimed to support PE within health care [39,40]. Studies exploring PE through the use of these types of bedside visual tools is also growing [[40], [41], [42], [43], [44], [45]].

One patient-provider communication whiteboard type of tool, the discharge bedside board (DBB), was developed at the Health Sciences Centre (HSC) Winnipeg, Canada. The aim of the tool, according to the organization's standard operating procedures (SOP), was to improve patient discharge planning and reduce length of stay through four strategies: 1) engaging patient and family in care and discharge planning; 2) identifying anticipated discharge date and or goals with family and the healthcare team, 3) improving care providers discharge planning and discharge communication at the bedside, and 4) providing an opportunity for patients and family to ask questions [46]. The ‘tool’ therefore includes the physical board and the processes related to board use. DBBs were typically 30″ wide and 20″ high and mounted near the patient's bedside, either on the adjacent wall, or beside the head of the bed. Some DBB were plain white and unstructured, while most units had boards that included pre-formatted labels for information such as today's date; name of nurse, health care aide and doctor; tests and appointments; patient and family questions; discharge goals; anticipated discharge date; and other care items. The DBBs had been initiated in 2010 and in 2015, a survey of the DBB had been conducted with nurses, physicians and allied health care providers to better understand and improve DBB use [47]. However, evaluation of its effectiveness in supporting PE in patient care and discharge planning lacked input from two key stakeholders – patients and their families. One strategy to strengthen assessment of communication tools is understanding stakeholders' perspectives. Their insights may reveal strengths and gaps in patient care and organizational practices that can inform tool-related revisions that may lead to more effective and patient-centered outcomes [26].

The objective of this study was to explore adult inpatient and family perspectives of a DBB for supporting their engagement in patient care and discharge planning to inform tool revision. Guided by the MFE [18], we sought to gain an understanding of participants' lived experiences related to the DBB, and the perceived barriers, facilitators and factors influencing the continuum of PE in supporting patient care and discharge. As researchers with a constructivist perspective [48] we acknowledge that our experiences as clinicians, clinical educators and researchers shape our understanding and interpretation of this work.

## 2 Methods

### 2.1 Design

A qualitative descriptive study using semi-structured interviews with key stakeholders at the patient bedside, and DBB-related photographic and SOP data was adopted. Qualitative descriptive designs are particularly suited to providing a summary of a phenomena in simple language reflective of that event, and when multiple data sources such as semi-structured interviews, observations, and documents are included [49].

### 2.2 Participants

Forty-five participant interviews were conducted (40 patient only, 4 patient and family, 1 family member only) with a purposive sample of English-speaking adult medical or surgical inpatients. Patient participant characteristics are presented in Table 1. Patients' family members were also invited to participate in the interview if present, and the patient agreed. In one case, the patient was unable to communicate, but a family member and legal guardian agreed to be interviewed. Family member participants included spouses and adult children. Patients were drawn from across seven medical and surgical care units to enhance maximum variation sampling [49] until the data appeared saturated. These units were identified for evaluation as they were the first units of the hospital to adopt the DBB as part of a discharge initiative and therefore reflected established use. The health facility, located in mid-western Canada, was a tertiary acute care center, that also served as an inner-city community hospital.

**Table 1:** Patient participant characteristics. (*n* = 44).

Variable	Total Patient Sample
Gender (n, %)	
Female	27 (61.4)
Male	17 (38.6)
Age in years (mean, SD)	58.5 (14.7)
Length of stay at interview in days (mean, SD)	14.3 (27.5)
Ethnicity (n, %)	
Canadian	18 (40.9)
French-Canadian	5 (11.4)
European	31 (70.5)
First Nations	3 (6.8)
Metis	2 (4.5)
Other	5 (11.4)
Missing	1 (2.3)

Note: Ethnicity self-reported as one or more of the above ethnic groups.

### 2.3 Patient and family interview guide

A brief semi-structured interview guide, developed by the research team, was used during interviews with patients and or family members. Interviewers recorded responses to questions exploring perceptions of DBB purpose, orientation, format, accuracy, currency, completeness, privacy, support for engagement in patient care and discharge planning, and suggestions for board improvement. Patient demographic information was invited. (See interview guide in Appendix A).

### 2.4 Procedure

Interviews with participants were held June – October 2017 following ethical review board and site impact approvals. Participants meeting study criteria were approached by a hospital staff to determine participation interest, and possible researcher follow-up to secure informed voluntary consent. One coffee card ($10) was given as an honorarium to acknowledge participant(s) time. Interviews were conducted at the patient's bedside by undergraduate (nursing, psychology, education) research assistants or Co-PI (DM) and ranged from 15 to 45 min (average 26 min) in duration. All interviewers were trained using the guide. Novice interviewers were paired with an experienced interviewer during data collection until skills were mastered. Interviews were conducted at the patient's bedside to support participants reflecting on the DBB and to enhance interviewer understanding of participant descriptions.

Participant responses were hand-written by the interviewer with responses and quotes confirmed during the interview and promptly transcribed by the interviewer to digital format and confirmed against interview records. Audio recordings while preferrable, were not feasible in order to prevent recording other patient or staff conversations since most patients shared rooms. Relocation to a private room was not possible due to limited patient mobility, care access requirements and no available nearby private space. Field notes were written to provide context. All patients and the legal guardian agreed to photographing of the DBB with the patient's name covered, for the restricted use by co-principal investigators (DM, DB) to inform analysis.

Study data included patient demographic information, transcribed interview responses, field notes, digital photographs of patient DBBs, and DBB SOP. Qualitative content analysis is a useful approach to analyze low inference visual and verbal data [49]. Through the identification of meaning units, condensing, coding and the generation of categories and themes [50] we created two visual fictitious summaries (DB, DM) of the photographic data (*n* = 45). A summary of the two-page SOP document was created, supporting study analysis.

While qualitative descriptive studies often use qualitative content analysis, the approach is amenable to multiple analytic methods [49], and both content and thematic analysis align with a perspective that data are variably accurate and truthful measures of reality [51,52]. For these reasons, and with an intent to provide a more detailed exploration of participant perspectives of their DBB experience, thematic analysis [[53], [54], [55]] was applied to the interview data, informed by field notes and photographic and SOP data. Table 2 provides the phases and processes taken in the thematic analysis. Final themes and quote selections were confirmed by all team members, including members with extensive clinical experience in the acute care context (DB, JP, GD) and expertise in qualitative methodologies (DM, KR). (See Table 3 for examples of the coding process.)

**Table 2:** Description of the phases taken in the thematic analysis of interview data.

Phase	Description of the study process
1. Familiarizing yourself with the data	Independent review (DB, DM) of transcribed interview data, informed by field notes, photographic and SOP data; reading and re-reading data and noting initial ideas.
2. Generating initial codes	Independent open coding (DB, DM) of all interview data using notes and memos; discussing initial codes and generating and applying common codes to all data using Word Table (DB) or Atlas.ti v8.2.4 (DM).
3. Searching for themes	Comparing and contrasting and collating codes into preliminary themes, mapping themes to related supporting data (DB, DM). Codes were not limited to specific interview questions, but rather were drawn from across the data. Groupings of themes were compared within and across participant interviews.
4. Reviewing themes	Confirming if themes fit with coded extracts and entire data set, reducing similar codes (DB, DM).
5. Defining and naming themes	Refining, defining, and naming of themes and subthemes (DB, DM) with team feedback on final themes, subthemes, and exemplars.
6. Producing the report	Final analysis (DM, DB) and development of the final report (DM with feedback from team)

Note: Analysis was based on the work by Braun and Clarke, 2006 [55].

**Table 3:** Examples of the thematic analysis processes for each of the four study themes.

Data extract	Initial codes	Preliminary Themes	Final Themes, Subthemes
Q. What do you think the board is for? *“For making sure that patients are aware of goals that are in place and for improving communications between the healthcare team, families, and team.”* (B4, Patient) *“It is to allow patients to know what is going on with their vital statistics and their day-to-day well-being. I was not told anything about the board when I came into the hospital”* (B9, Patient)	• Fair-good understanding • Patient-provider communication (1-way) • Family-provider communication (2-way) • Provider-provider communication • Limited understanding • Patient-provider information (1-way) • No recall of board orientation • Note. Sometimes no marker and or space to write questions (interviews, field notes, photographic data)	• Variable strength of understanding of board purpose • Multiple Communication Types o verbal orientation o board-based information o unspoken (e.g., no marker) • Variable targets, flows in communication	***Understanding the Board*** Subthemes: *Multimodal Communication: Visual and Verbal Synergy* *Multimodal Communication: Hit or Miss*
Q. What parts of the board do you like? *“I like that it gets updated often. You know what you are doing without asking.”* (B44, Patient) Q. Do you feel the board helps you to understand what needs to happen before you can get out of the hospital? *“Well, I know what I have to do before I get out, but it wasn't from the board…I think that should be up there.” (B30, Patient)*	• Format – current • Format - clarity • Information provides patient independence • Awareness of discharge plan • Discharge - board not currently helpful • Missing board information • Wanted more discharge information • Note. Frequent outdated or missing information, e.g., discharge plan, (field notes, photographic data)	Board information is valued for patient care/discharge planning Information value is contextual: currency, accuracy, clarity, presence	***Included Essential Information to Guide Care*** Subthemes: *Clinical Compass* *Support for Discharge…Maybe*
Q. What did you think of the amount of information on the board? *“I think it's just enough…I don't want everyone knowing too much information about why I'm here.” (B28, Patient)* Q. What do you think the board is for? *“I think what the board is for is a care plan… I don't like it, because it gives out too much information like my full name…It could be good for patients to see it, but I would suggest putting a cover on it to keep it private…I feel uncomfortable having others look at my business on the board.” (B20, Patient)*	• Public aspect of board information is a concern • Balance of information – too little, too much, just enough • Full name, patient ‘business’ in public view makes patient uncomfortable • Cover for board suggested • Note. SOP requires confirming with patient before writing personal information on the board. No DBB used bed number instead of patient name or ‘See Chart’ notation (*n* = 45, photographic data)	A fine balance of information Exposure is subjective and contextual Potential and real gaps in protocol related to privacy of information	***Balancing Information on the Board*** Subthemes: *Just Enough* *Current Threat of Exposure*
Q***.*** What do you think the board is for? *“It's supposed to be a two-way flow of information where healthcare professionals and family can communicate.” (B3, Patient)* Q. What parts of the board do you like? *“The nurse's name, categories if they're kept up to date (like tube feedings) …sometimes we use the board to help the nurses' with my daughter's care.” (B3, Family member)* *“Tells people that I am somebody, not just another patient…” (B21, Patient)*	• Board as a source of communication • Two-way flow of information between patient/family and care providers • Value of information – if current • Using the board to impact patient care • Patient value of information to tell providers – patient is a person • Note. SOP strategies support two-way communication for care and discharge through DBB	Connecting with and through the board Connection can be two-way Value of board is more than just information for the patient, but a way to value the patient	**Maintaining a Sense of Connection** Subthemes: *Engagement, a Two-way Flow* *Tells People that I am Somebody*

Lastly, integrated analysis of all study data, guided by the MFE [18], was performed exploring the continuum of PE related to the DBB within the direct care context.

Strategies to address the four criteria of trustworthiness of the study findings [56] were applied: 1) credibility (maximum variation and purposive sampling, data saturation, triangulation of data, confirmation of responses, team discussions on developing findings); 2) dependability (audit trail of procedures and analysis); 3) transferability (‘thick’ descriptions of participant experiences, visual DBB summaries); and 4) confirmability (code table and exemplars to demonstrate linkage from data to themes).

## 3 Results

**Fig. 1:**
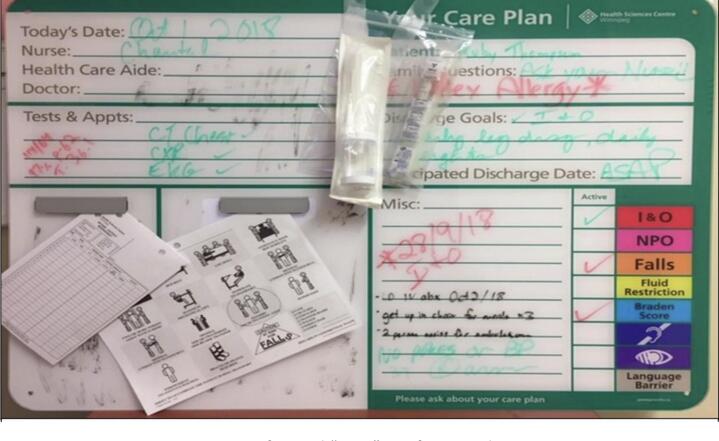
Fictitious representation of typical “poor” pre-formatted DBB.

**Fig. 2:**
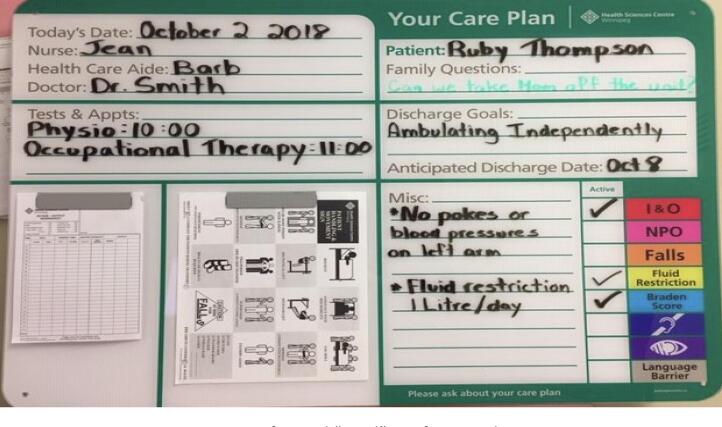
Fictitious representation of typical “good” pre-formatted DBB.

Two major white board formats were observed: unstructured (e.g., blank white board) and pre-formatted. Pre-formatted boards were the most common form (*n* = 35, 77.8%) observed, with four minor variations of pre-formatting noted. These variations included unit-specific differences in board labels, comment box location and care prompt details (e.g., I & O, turns). Resulting from the analysis (DM, DB) of the digital photographs, two fictitious mock-ups of pre-format board type were created based on identified recurring themes. Fig. 1 portrays a typical ‘poor’ pre-formatted board; Fig. 2 portrays a typical ‘good’ pre-formatted board.

‘Poor’ board characteristics included missing or outdated information; and information perceived as provider rather than patient-centered such as inclusion of multiple medical abbreviations, small, illegible, or ‘sloppy’ writing, pale marker coloring, clutter, including attached items (intake and output record sheets), items covering other information, and no writing space and or marker for patient or family use.

Conversely ‘good’ board characteristics included complete, current, legible, and neatly written and organized boards with room and marker available for family's questions. Even within the same pre-format, observed content and presentation was comparatively more patient-centered (e.g., clear, neat, colorful, unobstructed, simple language, no or few abbreviations based on photographic data) in boards evaluated more positively by participants. Although less common, unstructured DBB could be perceived as effective, depending on organization of content and legibility.

Four major themes were generated from the data: 1) Understanding the Board; 2) Included Essential Information to Guide Care; 3) Balancing Information on the Board; and 4) Maintaining a Sense of Connection. Themes and associated sub-themes are discussed in detail below followed by a synthesis of participant recommendations.

### 3.1 Theme 1: understanding the board

The first theme related to understanding the board based on visual and verbal communication. Participants appeared to interpret the purpose of the DBB and their role in engagement with it based on direct and indirect healthcare provider communication. Although SOP indicated the admitting nurse (or delegate) provide DBB orientation, most participants reported no or minimal recall of an orientation to the DBB. Some participants relayed they may have received orientation but reported no or poor recall due to illness or pain; high stress or anxiety; board not a priority at admission; and or memory problems. Two sub-themes related to multimodal communication were developed: *visual and verbal synergy*; and *hit or miss*.

#### 3.1.1 Multimodal communication: visual and verbal synergy

Participant perceptions of the board were more positive and they reported greater use of the tool if they recounted the following factors: effective DBB orientation or ongoing re-orientation and that staff regularly updated and referred to information on the board; *“I was told the plan and the nurse and I had discussed what will happen when she was writing stuff on the board…”(B26, Patient);* patient and family DBB communication was supported, such as through the availability of a marker and space to leave questions or a comment “ask your nurse” (*n* = 12; interview, field notes, and photographic data), and; the DBB design and content were ‘patient-friendly’, such as through neat, colorful and clearly visible writing, and the use of simple language, *“I like the way the board looks. It is very informative. Everything about the board is good. It is really colorful and attractive. The information on the board is pretty straight forward and easy to understand.” (B5, Patient; photographic data)* When communication elements aligned with SOP and worked synergistically, participants described the information and communication supported by the DBB as helpful.

#### 3.1.2 Multimodal communication: Hit or miss

For other participants, the effectiveness of the DBB to support PE appeared limited, sometimes reflecting potential gaps or inconsistencies in SOP application. Several participants reported that some visual and or verbal communication related to the board was omitted, including lack of a marker, or writing space (*n* = 7; interview, photographic data, field notes), and no orientation to the tool. *“I don't recall hearing anything.” (B11, Patient), “We weren't told we could use it.” (B17, Family member)*. Some patients believed information, such as DBB orientation was given but forgotten, *“Not a lot. I had other things on my mind.” (B16, Patient),* while others expressed that information on the board was inaccessible due to their visual challenges *“Hardest thing is to see it.” (B12, Patient)* and *“I can't read anything on the board. I don't have my glasses.” (B31, Patient).*

For the majority of participants, there were process and format elements related to the board that were perceived as being helpful, and other areas of gaps in communication. “*It is not too bad. I like a lot of things on the board like date, nurse, and doctor. I don't understand the information on the sides*” (e.g., NPO, I & O, turn Q2H, Braden Score) *(B9, Patient; no legend seen – photographic data)*. Other participants interpreted the board as only for staff, such as this patient *“I don't know what all that means. No one explained it to me – is it only for the staff?” (B26, Patient; missing date, names of nurse and doctor, included multiple abbreviations – photographic data),* and this family member *“The board is not patient friendly it's just meant for nurses.” (B18, Family member; no discharge goals or discharge date, attached ‘patient handling and movement sheet’ – photographic data).*

### 3.2 Theme 2: included essential information to guide care

The second theme that developed centered around the clinical impact of perceived essential information to support patient care and discharge planning related to the DBB, with sub-themes *clinical compass* and *support for discharge…maybe.*

#### 3.2.1 Clinical compass

The DBB served, to a variable degree, as a navigation tool, helping to orient patients and family members to the dynamic hospital environment, to predict and landmark short-term patient goals, and guide participants toward discharge. Understanding why and how to use this DBB and if the navigation tool was current, accurate, sufficiently detailed, and personally meaningful all influenced engagement and its effectiveness.

Up-to-date and accurate information such as staff names, date, care and discharge plans, and scheduled tests and appointments, in accordance with SOP, were especially valued and supported feelings of predictability, control and independence as conveyed by these two patients when asked ‘what parts of the board do you like’: *“I like the setup, especially the section where the appointments and tests are listed…I like knowing what's scheduled for me.” (B37, Patient; DBB appears current, complete – photographic data),* and *“I like that it gets updated often. You know what you are doing without asking.” (B44, Patient; DBB appears current – photographic data).*

When incomplete, the potential information was seen as valuable, but inaccessible, such as these patient and family participants, respectively, when asked ‘is there was anything that could be added to the board to make care better’; *“The nurse's names and the doctor's name” (B26, Patient; both names not listed – photographic data),* and *“We like the tests and appointment section, if it ever gets filled out.” (B18, Family member; date, nurse and doctor names listed but otherwise incomplete - photographic data).*

If the information presented was not provided in a way that was understandable, even patients who appeared interested in participating in their care were unsure of what action to take; *“The acronyms ‘I & O', ‘NPO'…What is the ‘Braden Score’? And what kind of ‘Turns' am I supposed to do?” (B24, Patient; multiple abbreviations, no legend – photographic data).*

#### 3.2.2 Support for discharge…maybe

Many participants believed the board identified helpful information as reflected by these three respondents when asked whether they thought the board supported discharge. One patient considered the discharge information helpful *“The ‘Discharge Goals’ and ‘Misc’ section has good information.” (B22, Patient; discharge goal and location complete, care notes listed - photographic data),* while two patients reported the DBB gave them more specific goals to support discharge: *“A short step is better than a stride. I'm aiming for those short steps.” (B1, Patient),* and *“The board made it very obvious I have to move around.” (B42, Patient; discharge goals list “mobilizing well” - photographic data).*

Other participants expressed limitations in the DBB specifically supporting discharge; some did not value the board as a source of discharge information, *“No, I believe the information on the board is more like information about day-to-day activities.”(B5, Patient),* and *“I wouldn't look at the board to seek information about any of that (discharge plan).”(B7, Patient).* While others saw only potential value, *“Well, I know what I have to do before I get out, but it wasn't from the board…I think that should be up there.” (B30, Patient),* who's response may have indicated a lack of understanding or agreement with listed discharge goals of *“off O2, resolution of shortness of breath”- photographic data).*

### 3.3 Theme 3: balancing information on the board

The third theme related to a need to balance type and amount of information presented on the DBB. Despite clear privacy-related procedures in the SOP, a potential or current concern for privacy was voiced in 47% (*n* = 21) of the interviews, with two sub-themes generated: *just enough*; and *current threat of exposure*.

#### 3.3.1 Just enough

Participants weighed the balance of having more or less information on the board. *“I think it's just enough…I don't want everyone knowing too much information about why I'm here.” (B28, Patient)* This balance was driven by type of information, context, and as expressed by one patient, personal preferences, *“That (level of detail) depends on the person. For me, it doesn't matter…for other people, they may be more private.”(B27, Patient)* Participants evaluated their desired information against privacy risk, *“I was thinking about them adding the Drug Protocol. But then, there is a privacy issue.” (B9, Patient).*

#### 3.3.2 Current threat of exposure

Despite SOP that care providers confirm patient agreement with information shared on the DBB, several participants expressed feeling exposed by the information available for other patients, visitors, or family to see when asked, ‘is there any information on the board that you feel should not be on there?’ *“I don't like it, because it gives out too much information like my full name…It could be good for patients to see it, but I would suggest putting a cover on it to keep it private…I feel uncomfortable having others look at my business on the board.” (B20, Patient, full name listed – field note)* While a greater DBB privacy concern arose in shared rooms, privacy threat was widely reported, “*Why are the tests on there? It should be private, and the family questions should also be private…We have family and friends visiting, and we don't want everyone knowing or scaring people into thinking that mom's condition is worse than it actually is.” (B18, Family member, patient full name listed - field notes).* It was notable, in reviewing the photographic data and field notes, that not one of the DBBs included the SOP prescribed use of bed number in place of patient name or the ‘See Chart’ notation in place of personal health information for patients uncomfortable having this information on the board, however, some boards did include notation to ‘ask the nurse’ if they had questions.

### 3.4 Theme 4. Maintaining a sense of connection

Participants saw the DBB as a tool for engaging in their own patient care, engaging with caregivers, and in offering hope and connection to the outside world. It could serve as a platform to empower connection between patients, family, and providers. It offered a mechanism to maintain a sense of humanity, such as through photos of pets or familiar landscapes or the personalized engagement by staff as they connected with patients through the DBB. Two subthemes emerged: *engagement, a two-way flow*; and *tells people that I am somebody*.

### 3.5 Engagement, a two-way flow

Compared to participants with poor or no recall of board orientation, participants who recalled an orientation to the board reported more communication with their care provider, a clearer understanding of the care and discharge plan, and confidence to write questions or communicate care issues. One family member had a clear understanding of DBB purpose, as illustrated here, “*It's supposed to be a two-way flow of information where healthcare professionals and family can communicate*.” *(B3, Family member; agreement with SOP).* This participant was unique in reporting using the DBB to direct patient care *“…sometimes we use the board to help the nurses' with my daughter's care.”* (B3, Family member).

#### 3.5.1 Tells people that I am somebody

In alignment with SOP goals to improve care providers' communication at the bedside, participants encouraged all care providers to engage with the board, including as a way to learn about and demonstrate valuing the patient. One patient described the significance of care providers connecting with the DBB in this way, *“It's important that medical staff are using it…before they approach… they should look at the board. This way the patient feels more important.”* (B8, Patient), while another patient stated that the DBB *“Tells people that I am somebody, not just another patient…”* (B21, Patient*).*

### 3.6 Participant recommendations

Participants offered recommendations on board format and processes. Overall, participants felt the DBB was a potentially useful communication tool needing some revision to be consistently effective to support PE in patient care and planned discharge. A compilation of patient and family recommendations with comparison to the organization's DBB expectations was generated to highlight congruences, potential gaps, and innovations and is found in Table 4.

**Table 4:** Participant recommendations compared to tool-related standard operating procedure.

Participant Recommendations	SOP	Other Observations
1. Provide clear verbal and written orientation and regular re-orientation to the board purpose and patient and family board engagement role.	General agreement.	Updates but not re-orientation included in SOP; no DBB handout* reported (interview data) or seen (field notes).
2. Ensure board design and content is patient and family centered. Use patient-friendly language, define acronyms with a legend, ensure writing and content is legible and neat, ensure patients can see the board (e.g., location, eyeglasses), provide space and markers for patient and family to flag communication requests.	General alignment to stated SOP aims and specifies provision of marker.	SOP does not comment on quality, legibility, or ability to see writing; SOP states staff are to ‘inform’ about various posted information.
3. Ensure that information on the board is complete and current.	Agreement.	
4. Be aware of threats to patient privacy. Confirm patient is comfortable with information on the board. Adopt private bedside electronic board, covers or sliding panels, private notes, or ‘flags’ to request private conversations in place of open question boxes, to reduce privacy threats.	Agreement; SOP included specific privacy strategies.	Participants offered additional board design suggestions to reduce privacy concerns.
5. Everyone should engage with the board – patient, family, and all staff. Staff should refer to the board prior to and during patient care.	General agreement.	SOP described use to improve bedside communication, participants specified use.
6. Support connection to the world and to maintaining a sense of personhood by including items such as weather, date, and space for patient interests such as photos of pets or pictures of home.	SOP includes date is minimally required.	Participants expanded the use of the DBB to provide a greater connection, than just to providers or care.

**Note:** *Discharge Bedside Board Standard Operating Procedure (SOP) indicated providing a patient and family handout on the DBB at orientation. While there was a draft record of this brief information sheet, no reports by participants or observations by research assistants indicated it had been given out during the data collection period, and feedback from clinical educator (DB) indicated that this aspect of the SOP was believed to have not been yet implemented, although this could not be confirmed with 100% certainty. For this reason, data did not include the handout document.

### 3.7 Integrated analysis of DBB study findings along the continuum of engagement

**Fig. 3:**
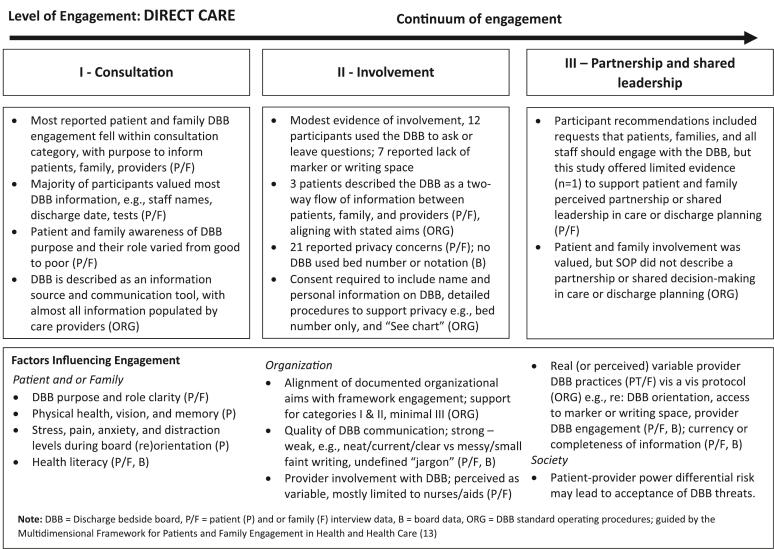
Exploring perceptions of the bedside discharge board in the context of the continuum of patient and family engagement.

Guided by the MFE, we explored study findings, situating the reported or observed DBB-related PE within the context of direct care and along a continuum of engagement, and identifying examples of significant factors influencing engagement within each of the three domains of patient and or family, organization, and society. As can be seen in Fig. 3, most reported PE fell within the consultation category, with only modest evidence of perceived involvement, and almost no evidence of partnership or shared leadership in care or discharge planning. By contrast, organization documentation aligned with both consultation and involvement, but lacked evidence to support PE at the most advanced level of engagement.

## 4 Discussion and conclusion

### 4.1 Discussion

Findings from our study with adult acute inpatients and family offer significant and novel insights into patient and family experiences and the influencing factors related to their continuum of engagement in patient care and planned discharge through the use of a DBB. Through the integrated exploration of multiple data sources and guided by the MFE [18] we were also able to situate the strengths and limitations of reported PE as related to this organizational tool, identifying perceived and observed gaps in DBB processes, and opportunities for targeted tool revision. Findings specifically add to understanding medical and surgical inpatient and family perspectives of PE related to DBBs, extending a growing body of research in PE in the acute care context [20,57].

Similar to other reports [30,40,43], a DBB can serve as a useful consultation level tool [45], conveying meaningful information to patients and family [58]. Notably, if information is not provided or current [40], or if visual accessibility [40,42] or health literacy level of information [26,59] is not patient-friendly, then the value of the PE tool, even as a simple one-way communication tool for supporting awareness of care or discharge information is significantly undermined.

Like reports in other studies of whiteboards, the DBB could support involvement in PE, such as the sharing of information [26,45], especially if purpose was understood [40,44] and markers and space were available [40]. The importance of effective tool orientation, re-orientation, and ongoing support for tool involvement cannot be overstated however, as without a clear understanding of board purpose, the tool can be interpreted as solely for staff or used only passively for information [44]. Barriers to PE when using the DBB, align with other reports of cognitive impact barriers to information strategies in acute care [26], and with general findings of cognitive deficits associated with patient factors such as pain, stress [60,61], fear, anxiety [27], age [61,62], surgery, grief, sedatives [61], mild traumatic brain injury [63], or sleep debt [64]. These findings underscore the need for care providers to be alert to potential PE barriers and to adjust communication based on unique patient care needs and information preferences.

Involvement level PE gaps in patient privacy related to DBB use are similar to those reported elsewhere [40], and provoke re-evaluation of this public format of communication. With the move for many organizations to electronic patient records, and the ability for patients to privately view and engage with these records, some privacy concerns reported here may not be as relevant elsewhere. Still, our findings and the continued use in many organizations of ‘publicly’ visible communication boards, emphasize the need for regular quality assurance of protocol application, and echo evidence that patients may not feel empowered to voice significant needs or concerns [26]. Although the patient-care provider dyad was not explored in this study, strategies supporting PE through therapeutic alliance [17] between patients and their care providers may help reduce barriers negatively impacting involvement in care and discharge planning decisions.

Partnership and shared leadership between patients and health professionals at the direct care level [18] is an important goal and reflective of the most advanced level along the PE continuum. However, gaps found here and reported elsewhere [40] reflect a troubling disconnect between organizational aims for PE and patients' lived experiences. Successful PE at this advanced level appears to hinge on strong organizational and professional commitment throughout an organization to be successful [39], and the acknowledgement that supporting inpatients and their family members to be full partners in care will require complex interventions and shifts in power between patients, providers and health care organizations (p. 9–10) [57].

The study offered both strengths and limitations. The sample explored views from a diversity of adult inpatients from multiple units, and with experience with different white board formats, but was limited to patients on medical and surgical care units. The sample included some ethnic diversity but under-represented non-English speaking patients, who may be at greater risk for engagement challenges. The inability to digitally record interviews may have led to some data loss. Patient and family perspectives were enriched by photographic data and comparisons to organization based DBB documentation. While study data offered both perceived and objective data related to the DBB, our findings offer only a partial appraisal of PE related to this tool. Future research exploring the perspectives of all key stakeholders (patients, family, care providers) and their interaction with the tool and each other are recommended.

### 4.2 Innovation

Guided by the MFE [13], we performed a novel integrated analysis of multiple DBB-related data. Based on patient and family reports, field notes, photographic records of tool use, and an organizational DBB procedural document, analyses enabled situating the findings along a continuum of PE and identifying factors impacting engagement.

### 4.3 Conclusion

This qualitative descriptive study offers helpful perspectives on the lived experiences of inpatients and their families on the use of DBB to enhance patient care and discharge planning. Findings underscore the importance of congruent PE goals and actions across the patient-family-provider-organizational system to optimally support effective care and discharge outcomes. By exploring patient and family perspectives and related DBB data, strengths and opportunities were identified, informing targeted revisions toward tool application.

## Ethical considerations

The study was approved by the Ethical Review Board at the University of Manitoba, no. E2016:107;HS20118. Before the interviews, participants were given written and verbal information about the study and informed that participation was voluntary and could be withdrawn at any time. All participants signed a consent form. We confirm all patient and personal identifiers have been removed or disguised so the patient/person(s) described are not identifiable and cannot be identified through the details provided.

## CRediT authorship contribution statement

**D.E. McMillan:** Conceptualization, Methodology, Funding acquisition, Supervision, Formal analysis, Writing – original draft, Writing – review & editing. **D.B. Brown:** Formal analysis, Validation, Writing – review & editing. **K.L. Rieger:** Methodology, Validation, Writing – review & editing. **J. Plouffe:** Conceptualization, Funding acquisition, Validation, Writing – review & editing. **G. Duncan:** Validation, Writing – review & editing. **C.C. Amadi:** Investigation, Data curation, Validation, Writing – review & editing. **S. Jafri:** Investigation, Data curation, Validation, Writing – review & editing.

## Funding

This work was generously supported by the 10.13039/100008252Health Sciences Centre Foundation.

## Declaration of Competing Interest

The authors declare the following financial interests/personal relationships which may be considered as potential competing interests:

Gregory Duncan reports a relationship with Health Sciences Centre Winnipeg that includes: employment. Corresponding author is the Clinical Chair at Health Sciences Centre, Diana McMillan; Co-authors were previously employed at Health Sciences Centre, Devon Brown and Jannell Plouffe.

## Appendix A Supplementary data

Supplementary material Image 1
