# Supplementary material for: Patient and family perceptions of a discharge bedside board
Source: PEC Innov. 2023 Sep 11;3:100214. doi: 10.1016/j.pecinn.2023.100214 (PMC10514555; doi:10.1016/j.pecinn.2023.100214)
Supplement: Supplementary file 1 — Supplementary material [file mmc1.docx]

**APPENDIX A**

**SEMI-STRUCTURED INTERVIEW GUIDE**

**Introduction to the Interview:**

Thank you for agreeing to meet with me. I would like to learn more about your thoughts and experience around the use of the bedside board. I would like you to share your experience with the board from admission to the present time. I also want to inform you that you do not have to answer a question if it makes you uncomfortable. All information shared will be kept confidential and you may withdraw from the study at any time. In order to document your responses, I will take notes during our conversation. Do you have any questions before we begin?

1. **What do you think the board is for?**

- What were you told about the board when you came into hospital? (Prompt example)
- What were you told about the board when your family member came into hospital? (If family present)

1. **What are your thoughts on the way the board looks?**

- What parts of the board do you like? What parts don’t you like?
- Is there any information on the board that you cannot understand? Or that does not make sense? If so, what?
- Is there any information on the board that you cannot read? If so, why?
- What did you think of the amount of stuff /information on the board?
- Was there any information that is important to you missing from the board?

1. **Is there any information on the board that you feel should not be there? If so, what?**
2. **Does the board have the information needed to help you or your family in planning to get you out of hospital?**

- If so, what information did you find helpful? What information was the most valuable?
- What could be added to the board to help you and your family in planning to get you out of hospital?
- Looking at this board right now, do you feel it helps you understand what needs to happen before you can get out of hospital? If no, what is missing?

1. **Did you feel the board reflected what you think needs to be done to get you out of the hospital?**
2. **What do you feel could have been added to the board that would make your care better?**
3. **How much detail would you like to see on the board?**
4. **How often was the board being updated? Is the information current or correct or accurate?**
5. **Can you tell me about a time when the information on the board was not right?**
6. **Were you or your family able to use the board to ask questions of staff? If yes, how? What questions did you ask and were they answered?**
7. **Do you have anything else you’d like to add to make the boards better?**
8. **How many days have you (your family member) been in hospital for?**
9. **What is your (your family member) age: _________**
10. **What best describes the ethnic or cultural group(s) to which you (your family member) belong?**
